# Tuning electronic structure and carrier transport properties through crystal orientation control in two-dimensional Dion-Jacobson phase perovskites

**DOI:** 10.1186/s40580-024-00473-y

**Published:** 2025-01-13

**Authors:** Byunggeol Kim, Jeehong Park, Donghee Kang, Na Eun Jung, Kitae Kim, Hongsun Ryu, Joon Ik Jang, Soohyung Park, Yeonjin Yi

**Affiliations:** 1 Department of Physics, Yonsei University, Seoul, 03722 Republic of Korea; 2 Advanced Analysis & Data Center, Korea Institute of Science and Technology (KIST), Seoul, 02792 Republic of Korea; 3 Department of Physics, Sogang University, Seoul, 04107 Republic of Korea

**Keywords:** 2D perovskite, High orientation, Crystal, Electronic structure, Carrier mobility

## Abstract

Two-dimensional halide perovskites are attracting attention due to their structural diversity, improved stability, and enhanced quantum efficiency compared to their three-dimensional counterparts. In particular, Dion-Jacobson (DJ) phase perovskites exhibit superior structural stability compared to Ruddlesden-Popper phase perovskites. The inherent quantum well structure of layered perovskites leads to highly anisotropic charge transport and optical properties. Therefore, controlling the preferred crystal orientation (parallel or perpendicular) is crucial for optimizing device performance. This work presents a rational strategy to control parallel and perpendicular crystal growth in C_6_N_2_H_16_PbI_4_ (4AMPPbI_4_)-based DJ phase perovskite thin films. We demonstrate that crystal orientation depends on crystal growth rates, which can be controlled by varying the solvent composition, antisolvent, and annealing temperature. Direct and inverse photoelectron spectroscopy reveals that the electronic structure of 4AMPPbI_4_, including its work function, ionization energy, and electron affinity, is orientation-dependent. Different orientations significantly affect carrier transport as confirmed by single-carrier devices. This study highlights the critical role of crystal orientation in DJ phase perovskites for designing high-performance optoelectronic devices.

**Graphical Abstract:**

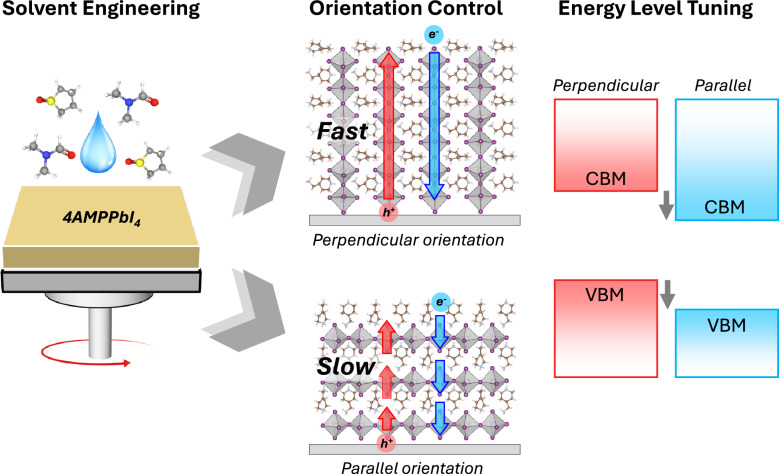

**Supplementary Information:**

The online version contains supplementary material available at 10.1186/s40580-024-00473-y.

## Introduction

The organic–inorganic halide perovskites have been extensively studied due to their exceptional optoelectronic properties, including high absorption coefficients [1–3], high carrier mobilities [4, 5], small exciton binding energies [6, 7], narrow emission wavelengths, and high photoluminescence quantum yields [8–10]. These characteristics have propelled perovskites to the forefront of research in optoelectronic device applications such as photovoltaics [11–13], light-emitting diodes [14–16] and photodetectors [17, 18].

Two-dimensional (2D) perovskites with anisotropic structures have garnered interest in optoelectronics due to their tunable properties. By controlling the organic spacer between metal halide octahedra, researchers can tailor structural diversity, improve stability, and enhance quantum efficiency [19–21]. Generally, the anisotropic nature of materials, particularly crystal orientation, significantly influences their physical properties, including electronic and structural characteristics. For instance, carrier mobility in conjugated polymers can be enhanced by approximately 100 times through improved structural anisotropy and stronger π-π interactions [22]. Furthermore, numerous studies have demonstrated that manipulating the orientation of organic molecules can alter their intrinsic ionization energy, impacting carrier injection [23–25]. While perpendicularly oriented crystals in Ruddlesden-Popper (RP) phase 2D perovskites have been suggested to enhance optoelectronic device efficiency due to the planar arrangement of [PbI_6_]^4–^ octahedra facilitating efficient carrier transport [26–28], recent attention has focused on Dion-Jacobson (DJ) phase 2D perovskites, exhibiting superior structural stability compared to their RP counterparts [29, 30]. In particular, Shang et al. reported that perovskite LEDs utilizing DJ structures exhibit a stability of approximately 100 h (T_50_), which is nearly two orders of magnitude greater than that of LEDs with RP structures [31]. The influence of crystal orientation (parallel vs. perpendicular) on carrier transport in DJ phase perovskites is likely more subtle: The stronger hydrogen bonding between organic and inorganic layers in DJ phase perovskites compared to RP phase perovskites [32] suggests that a more complex relationship between orientation and carrier transport. Further investigation is needed to fully elucidate this relationship.

This study investigated the impact of crystal orientation on the electronic structure and carrier transport properties of C_6_N_2_H_16_PbI_4_ (4AMPPbI₄; 4AMP = 4-(aminomethyl)piperidinium) perovskite thin films. 4AMPPbI₄ was selected due to its extensive characterization in previous studies [33–35], making it a suitable candidate for exploring orientation-dependent properties in DJ phase 2D perovskites. Uniform thin films with high crystallinity and controlled parallel and perpendicular orientations were fabricated by adjusting the solvent composition and processing temperature. Photoelectron spectroscopy (PES) analysis revealed that the electronic structure of 4AMPPbI₄ is indeed orientation-dependent, suggesting potential variations in carrier behavior. To investigate this further, hole-only devices (HODs) and electron-only devices (EODs) were fabricated with both parallel and perpendicularly oriented perovskite layers. These devices employed a sandwich-type architecture with top and bottom electrodes. The current density–voltage characteristics revealed enhanced carrier mobilities/transport in devices with perpendicularly oriented perovskite layers. This enhancement is attributed to the formation of efficient carrier transport pathways facilitated by the perpendicular arrangement of [PbI_6_]^4–^ octahedra, which directly connect the top and bottom electrodes.

## Results and discussion

### Control of thin film orientation and fabrication method

**Fig. 1:**
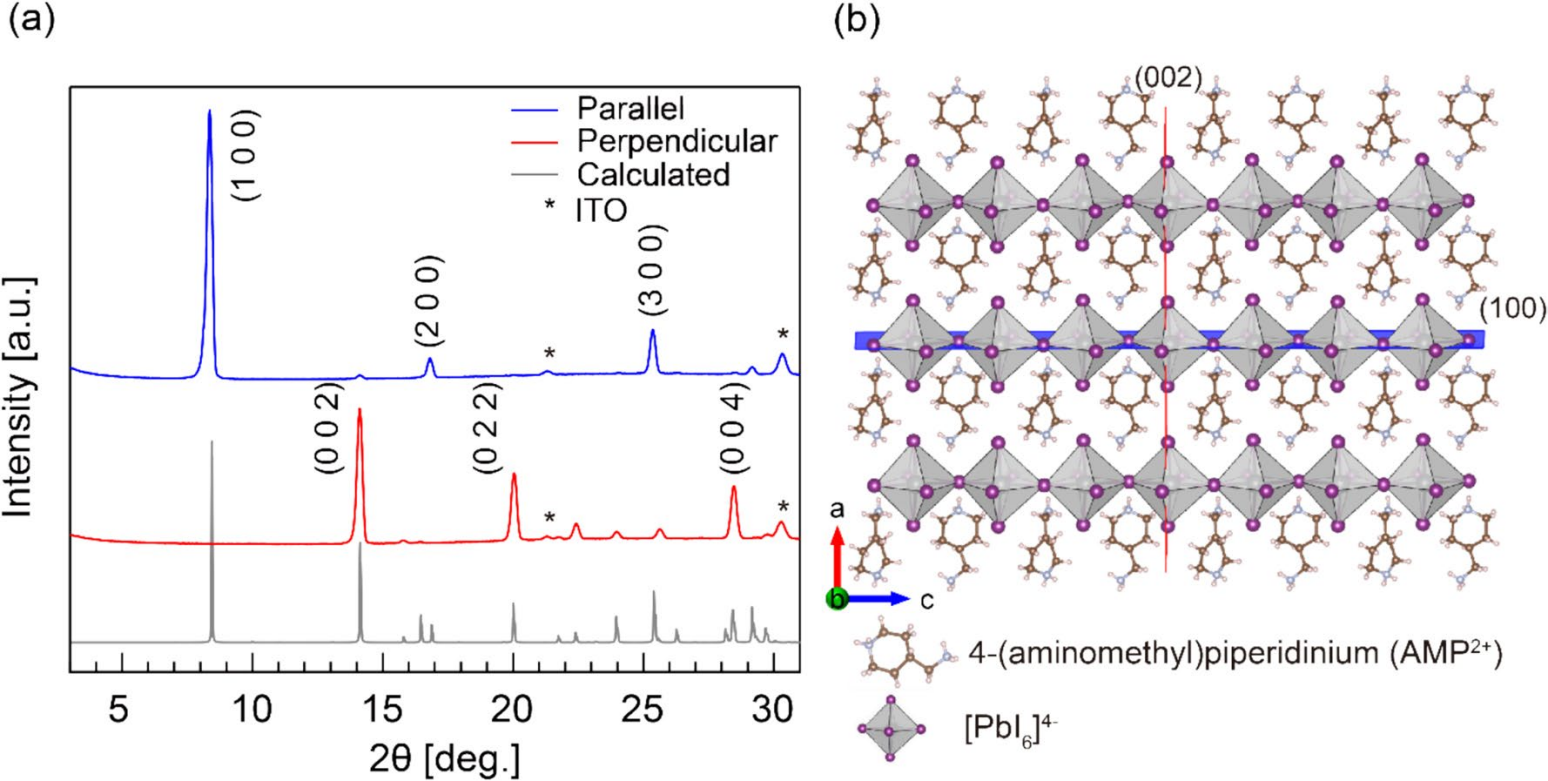
**a** XRD patterns of DJ phase 2D 4AMPPbI₄ (n = 1) perovskite thin films and (**b**) schematic crystal structures illustrating parallel (100) and perpendicular (002) orientations with respect to the substrate plane

Figure 1 shows the XRD patterns and schematic crystal structures of 4AMPPbI_4_ perovskite thin films with different preferential crystal orientations on indium tin oxide (ITO) substrates. As shown in Fig. 1a, the top XRD pattern (blue line) exhibits distinct peaks at 2θ = 8.4°, 16.9°, and 25.5°, corresponding to the (100), (200), and (300) reflections, respectively, of the parallel-oriented 4AMPPbI_4_ with n = 1 phase. The middle XRD pattern (red line) shows peaks at 2θ = 14.1° and 28.4°, corresponding to the (002) and (004) reflections of the perpendicularly oriented 4AMPPbI_4_ phase. The interlayer distance of 10.52 Å and the in-plane pseudo-cubic lattice parameter of 6.27 Å, obtained from Bragg's law, are consistent with literature values [32]. Notably, the parallel-oriented film exhibits high phase purity, and the perpendicularly oriented film shows a minor (022) phase mixed with the dominant (002) phase.

Figure 1b illustrates the crystal structures. In the parallel orientation (100), the [PbI_6_]^4⁻^ octahedral cages are aligned parallel to the substrate, forming a sandwich-like configuration with AMP cations inserted between the [PbI_6_]^4⁻^ planes. Conversely, the perpendicular orientation (002) features both [PbI_6_]^4⁻^ planes and AMP organic spacers aligned perpendicular to the substrate. These results demonstrate the successful growth of highly oriented 4AMPPbI₄ perovskite films with controlled parallel and perpendicular orientations.

To fabricate orientation-controlled perovskite thin films, we developed a novel method. While several techniques for obtaining high-quality perovskite films have been reported, involving precise control of the solvent type and ratio, antisolvent choice [36], annealing temperature [37, 38], and other parameters [39, 40], we found that conventional methods employing DMSO and DMF solvent combinations yielded poor crystallinity and morphology (Figure S1). Therefore, we prepared perovskite solutions by dissolving 4AMPPbI₄ crystal (thus perfect stoichiometric) powder in a carefully chosen combination of THTO and DMF. Controlling crystal orientation requires careful regulation of the crystal growth rate and environment temperature. Previous reports have indicated that THTO exhibits a stronger interaction with Pb^2+^ ions compared to DMSO and DMF, stabilizing Pb^2+^ and influencing crystal growth direction and, consequently, orientation [41, 42]. By adjusting the THTO ratio, we can therefore manipulate the crystal orientation.

**Fig. 2:**
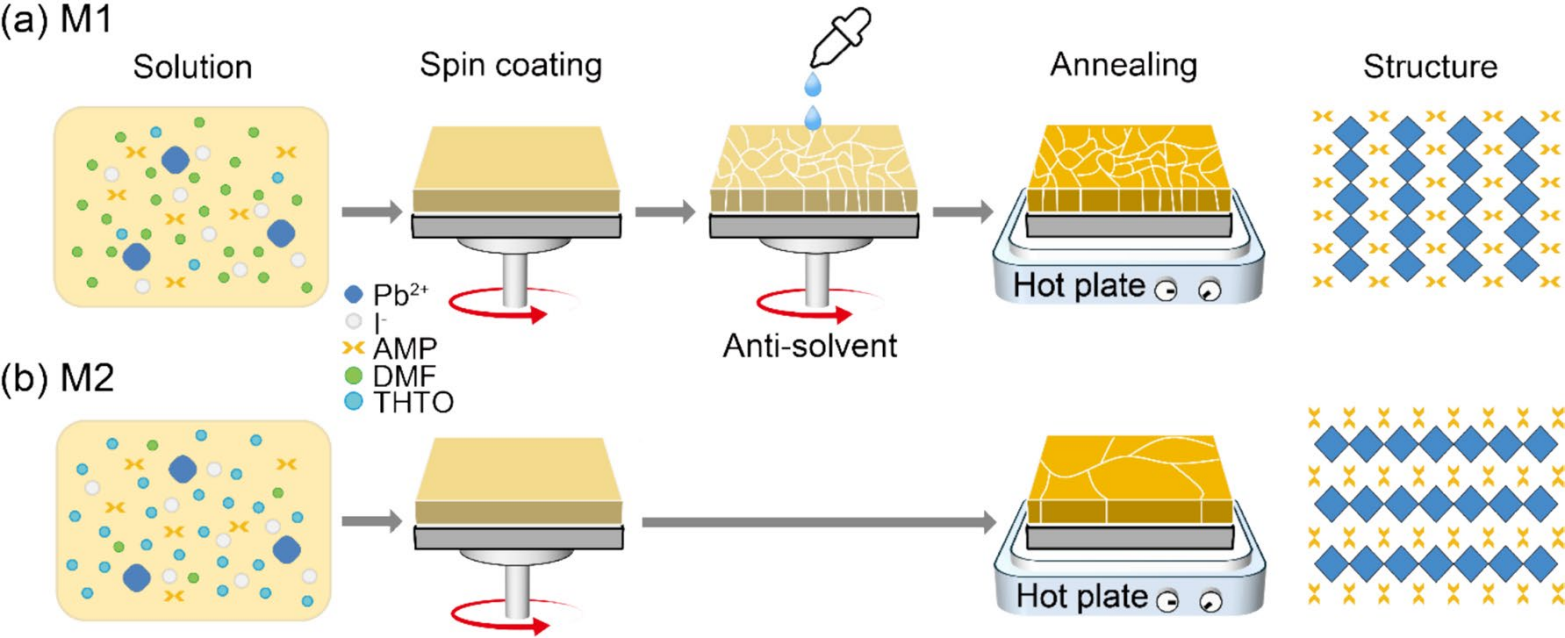
Schematic diagram of the crystal growth process of perovskite films with (**a**) M1: perpendicularly oriented crystal and (**b**) M2: parallel oriented crystal. All films were spin-coated on ITO substrates

Figure 2 illustrates the unique crystal growth processes developed to achieve high-quality 4AMPPbI₄ perovskite films with controlled crystal orientations. Perpendicularly oriented films (a) were fabricated using Method 1 (M1), which combines the conventional antisolvent method with precise control of the DMF:THTO solvent ratio in the perovskite solution, followed by stepwise annealing. Various antisolvents were tested, and chlorobenzene (CB) was found to yield the best film morphology (Figure S2). Parallel-oriented films (b) were prepared using Method 2 (M2), which relies solely on controlling the DMF:THTO ratio without antisolvent, followed by a single-step annealing. XRD analysis results revealed that the formation of (100) plane indicating a parallel direction was promoted when the THTO ratio was high (Figure S3). To optimize the orientation and crystallinity of the 4AMPPbI_4_ films, both the DMF:THTO ratio and annealing temperature were carefully controlled for each method.

**Fig. 3:**
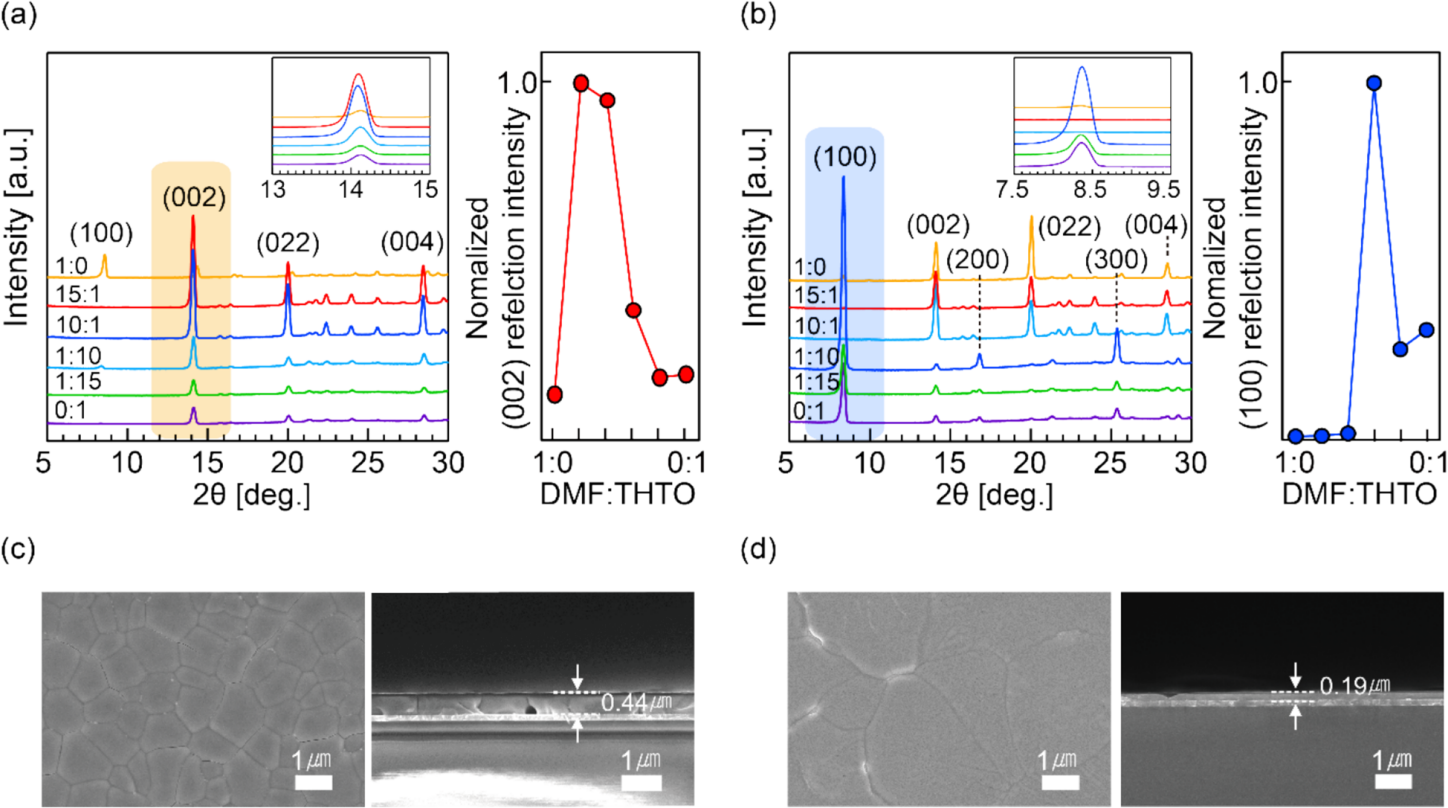
X-ray diffraction (XRD) patterns of (**a**) perpendicularly oriented 4AMPPbI_4_ films and (**b**) parallel-oriented 4AMPPbI_4_ films fabricated with various DMF:THTO ratios. Surface morphology and cross-sections of 4AMPPbI_4_ films with (**c**) perpendicular orientation and (**d**) parallel orientation

The crystal orientations resulting from different DMF:THTO ratios in the M1 and M2 processes were analyzed using XRD, and the corresponding surface morphologies were characterized by SEM and optical imaging (Figures S4 and S5). Figure 3a shows the XRD patterns for films fabricated using the M1 process with CB as the antisolvent. The annealing temperature was ramped in four steps (70 °C, 110 °C, 150 °C, and 180 °C) to induce perpendicular orientation. When THTO is used as the sole solvent, only a very weak (002) peak is observed, indicating minimal perpendicular orientation. As the DMF content increases, the intensity of the (002) peak increases significantly. However, using only DMF results in the appearance of a (100) peak alongside the (002) peak. Optimal perpendicular orientation with high crystallinity is achieved in the M1 process with a small amount of THTO (DMF:THTO = 15:1). Since parallel-oriented crystals could not be obtained using the M1 process, the M2 process was employed, which involves single-step annealing at 180 °C without an antisolvent. Figure 3b shows the XRD patterns of films fabricated using the M2 process with varying DMF:THTO ratios. In contrast to the M1 process, the highest peak intensity at 2θ = 8.4° (corresponding to the (100) reflection) is observed under THTO-rich conditions (DMF:THTO = 1:10), indicating strong parallel orientation.

Figures 3c, d show surface and cross-sectional SEM images of films with perpendicular and parallel orientations, respectively, fabricated under each optimized condition. The perpendicularly oriented film grown using the M1 process exhibits a smaller grain size (c), while the parallel-oriented film grown using the M2 process shows a larger grain size and lower thickness (d). These morphological differences provide valuable insights into the orientation-dependent crystallization mechanisms discussed below.

### Proposed mechanism of orientation-dependent crystal growth

Our results demonstrate that various factors, including precursor solvent, annealing temperature, and antisolvent, significantly influence the crystallization and orientation of 4AMPPbI₄ thin films. Understanding the interplay of these factors is crucial for controlling crystal growth and orientation. Several mechanisms governing crystal orientation in thin films have been proposed in previous studies [43–45]. Based on these studies and our experimental observations, we have deduced a mechanism to explain the growth of 4AMPPbI_4_ crystals with parallel and perpendicular orientations.

**Fig. 4:**
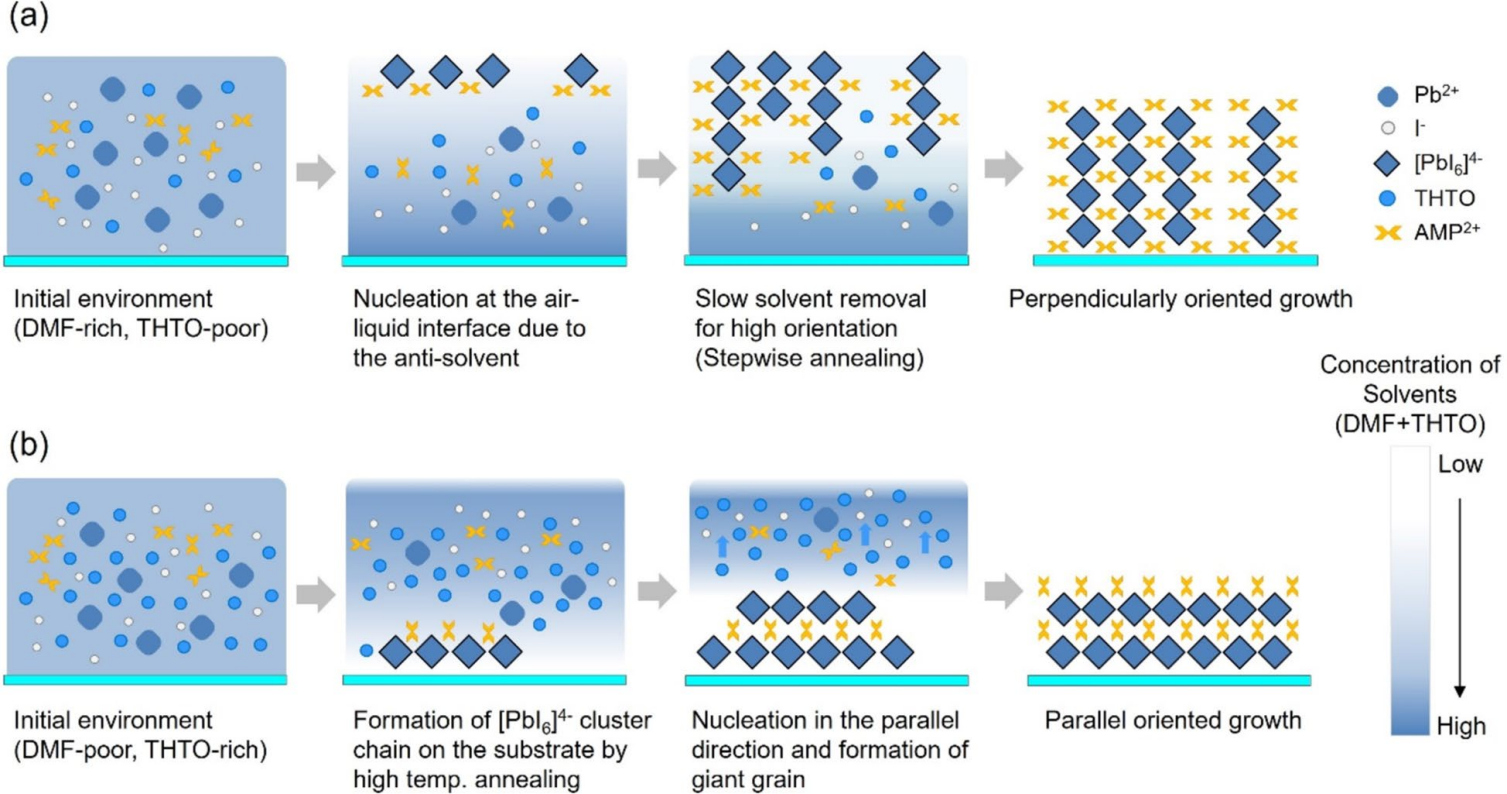
Schematic illustration of the proposed mechanism for M1 and M2 processes. **a** M1: Low THTO content and the use of antisolvent promote rapid nucleation at the air–liquid interface and slow solvent removal, leading to perpendicular orientation. **b** M2: High THTO content facilitates parallel-oriented nucleation and crystallization due to the stronger THTO-Pb^2+^ interaction

Figure 4 schematically depicts the proposed mechanism of crystal growth for 4AMPPbI₄ perovskite in the M1 and M2 processes. In the precursor solution, Pb^2^⁺ ions interact with the lone pair electrons of both DMF and THTO. However, as previously mentioned, Pb^2^⁺ ions exhibit a stronger interaction with THTO compared to DMF. Therefore, the film growth mechanism can be manipulated by adjusting the ratio of these two solvents as well as THTO is a key to induce high orientation by slow crystallization [41].

The M1 process, illustrated in Fig. 4a, is expected to induce the perpendicular orientation of 4AMPPbI_4_ perovskite on the ITO substrate. This process involves a low THTO content and the use of an antisolvent. With excess DMF, the Pb^2+^ ions in the precursor solution predominantly interact with the lone pair electrons in DMF rather than THTO. Consequently, initial [PbI_6_]^4–^ chains/planes are readily formed in DMF-rich solvents due to the relatively weak interaction between DMF and Pb^2+^ ions compared to THTO. Furthermore, the use of an antisolvent creates an oversaturated zone with a high concentration of precursor species at the air–liquid interface. This zone acts as a nucleation site, promoting rapid nucleation around the [PbI_6_]^4–^ chains/planes. Subsequent crystal growth proceeds vertically from these nuclei during annealing, leading to the formation of grain boundaries between crystal groups that develop around the interface. This growth behavior is similar to that reported by Chen et al. for vertically oriented 2D perovskite (BA_2_MA_3_Pb_4_I_13_) [43], where nucleation and growth occur at the liquid–air interface. Following stepwise annealing further enhances crystal orientation. (Figure S6).

In contrast, the M2 process for parallel-oriented crystal growth can be described as a conventional method for 2D perovskites with n = 1 [46, 47]. In the RP phase compound (BA)_2_(MA)_n-1_Pb_n_I_3n+1_, parallel growth is prominent when n = 1, corresponding to a monolayer perovskite. However, for n > 1, increased perovskite layer thickness leads to competition between BA and MA ions, resulting in mixed-orientation growth [48, 49]. Similarly, the DJ phase 4AMPPbI_4_ (n = 1) in this study exhibits a strong preference for parallel growth due to the absence of competing precursors. This inherent preference for parallel growth is further enhanced by the optimized fabrication process. As shown in Fig. 4b, the high THTO content in the precursor solution strengthens the interaction between Pb^2+^ ions and the sulfoxide group of THTO, slowing down the nucleation process. Furthermore, the absence of an antisolvent prevents the formation of an oversaturated zone at the air–liquid interface. This slow crystallization promotes the preferential formation of [PbI_6_]^4−^ planes due to the stronger bonding tendency of the inorganic components than the organic counterparts. Organic spacers are then incorporated, forming 2D multilayer films and thus promoting parallel growth. Subsequent high-temperature annealing further enhances the morphology and crystallinity of the film.

### Electronic structure and carrier behavior

**Fig. 5:**
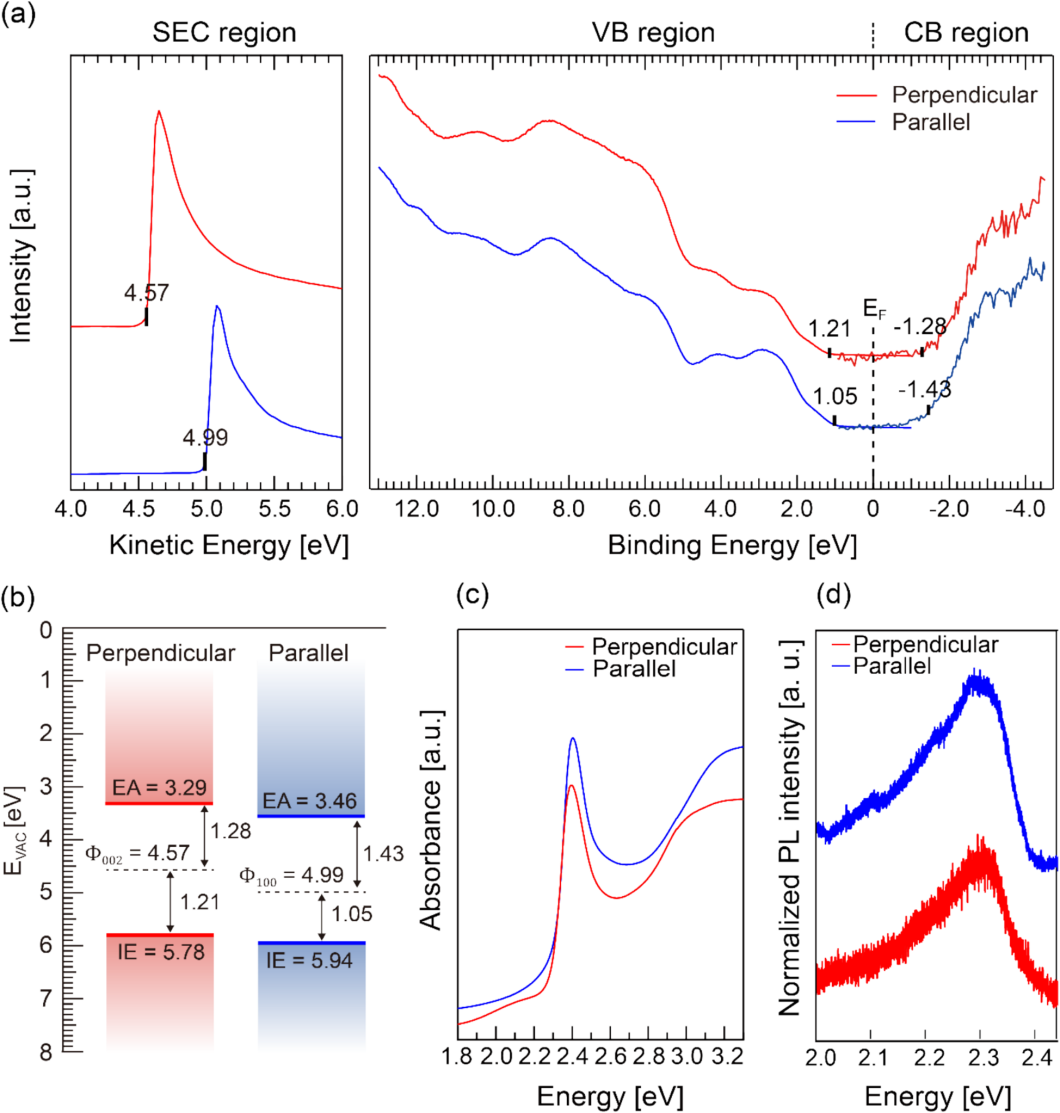
**a** UPS and IPES spectra of 4AMPPbI_4_ films having different orientation. SEC, valence and conduction band regions are presented. **b** Energy-level diagrams deduced from the spectra. **c** UV–Vis spectra and (d) Photoluminescence spectra of both orientation

To investigate the impact of crystal orientation on the electronic structure, ultraviolet photoelectron spectroscopy (UPS) and inverse photoelectron spectroscopy (IPES) measurements were performed, as shown in Fig. 5a. The estimated work function, ionization energy (IE) and electron affinity (EA) are summarized in Fig. 5b. Our combined UPS/IPES measurements reveal that electronic band gaps (*E*_g_) are nearly identical for both orientations (2.48–2.49 eV). These values are essentially identical as expected since the band gap is an intrinsic material property. Similarly, optical absorption and photoluminescence measurements show no discernible differences between the two orientations as shown in Fig. 5c, d. For both samples, we observe a strong excitonic transition centered at 2.4 eV followed by a monotonically increasing inter-band absorption in Fig. 5c. Photoluminescence (PL) spectra in Fig. 5d show a Stokes shift of ~ 100 meV with an additional tailing at lower photon energy, of which the origin is not still clear. Having identified the *E*_g_ from the UPS/IPES measurements and excitonic transition energy (*E*_exc_) from the optical absorption measurements, we determine the exciton binding energy (*E*_b_) of 4AMPPbI_4_ to be 80 meV and 90 meV for sample oriented parallel and perpendicular to the substrate, respectively. The binding energy is calculated using the following equation, ${E}_{\text{b}}= {E}_{\text{g}}- {E}_{\text{exc}}=\left(IE-EA\right)-{E}_{\text{exc}}$. Notably, the measured exciton binding energy of 4AMPPbI_4_ lies between the values reported in three-dimensional (3D) perovskites (e.g., 2–60 meV for CH_3_NH_3_PbI_3_) and RP phase 2D perovskites (e.g., 223 meV for PEA_2_PbI_4_) [50–52]. The intermediate *E*_*b*_ in 4AMPPbI_4_ indicates that confinement effects, either via dielectric- or quantum- confinement, further increase the binding energy compared to their 3D counterparts, but not to the extent in RP type perovskites.

While both the transport and optical band gaps remain almost unchanged, a clear distinction in absolute energy level positions is observed when referencing the vacuum level for each orientation. Specifically, the ionization energy (IE) and electron affinity (EA) are 0.16 eV larger in a film with parallel orientation (Fig. 5b). The energy separation between frontier orbitals (valence band maximum and conduction band minimum) and core levels for all elements remains the same for both orientations (See figure S7 for core-level XPS spectra). This observation indicates that the difference in absolute energy level originates from variations in the local vacuum level positions, which are often influenced by the film orientation [23, 53].

The differences in absolute energy level positions in Fig. 5b may indicate that hole injection is favored in perpendicularly oriented films, while electron injection is favored in parallel-oriented films. However, overall charge transport is not solely determined by charge injection; the dominant charge conduction pathway (interlayer transport for parallel orientation vs. intralayer transport for perpendicular orientation) also plays a crucial role. To investigate this aspect further, single-carrier devices were fabricated, and their mobility characteristics were analyzed.

**Fig. 6:**
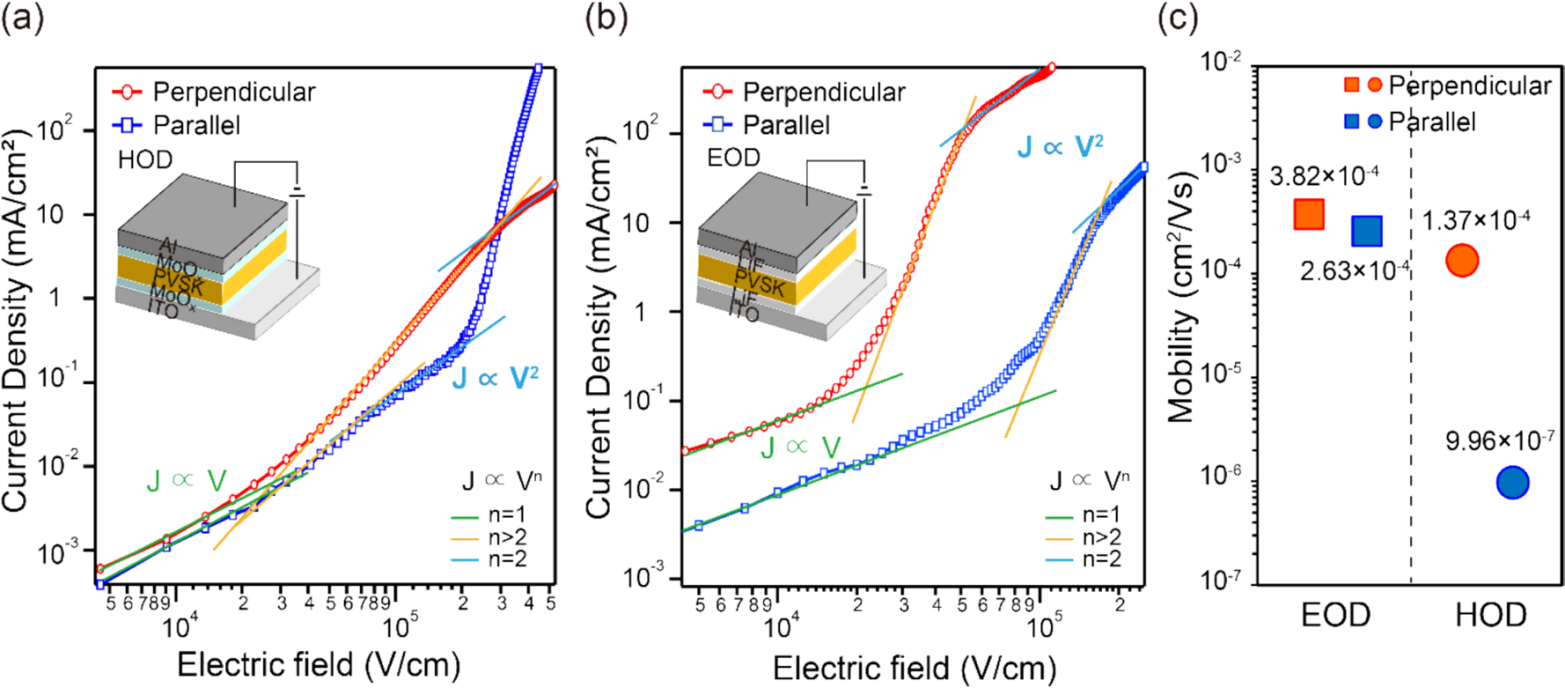
Current density–electric field curves from (**a**) hole-only devices and (**b**) electron-only devices with perpendicular and parallel orientations and (**c**) their mobilities

Hole-only devices (HODs) and electron-only devices (EODs) were fabricated and characterized, as shown in Fig. 6. The cross-sectional structures of the devices were characterized by SEM (Figure S8). To explore carrier behavior, the devices were characterized by measuring dark current density (J) as a function of electric field (E), as shown in Fig. 6. To eliminate the influence of device-specific factors such as charge injection barriers and contact resistance from electrodes, we focused on the intrinsic carrier mobility within the perovskite films, which was evaluated by fitting the space-charge-limited current (SCLC) region of the current density–voltage characteristics to the Mott-Gurney law [54, 55],

$$J=\frac{9}{8}{\varepsilon }_{r}{\varepsilon }_{0}\mu \frac{{E}^{2}}{L} \left(E= \frac{V}{L}, J\propto {V}^{2}\right),$$

where *ε*_*0*_ is the vacuum permittivity (8.854 × 10^−12^ F m^−1^), *ε*_*r*_ is the relative dielectric constant of 4AMPPbI_4_, *μ* is the carrier mobility, *E* is the electric field, and *L* is the thickness of the perovskite layer. The dielectric constant of 4AMPPbI₄ (ε_r_ = 5.7) was estimated from the correlation equation of the exciton binding energies of 2D and 3D perovskites [56, 57]. (See Supplementary Material, Table S1 for details).

The calculated carrier mobilities are shown in Fig. 6c. For the parallel-oriented sample, the hole and electron mobilities are μ_h(100)_ = 9,96 × 10⁻⁷ cm^2^/Vs and μ_e(100)_ = 2.63 × 10⁻^4^ cm^2^/Vs, respectively. For the perpendicularly oriented sample, the corresponding values are μ_h(002)_ = 1.37 × 10⁻^4^ cm^2^/Vs and μ_e(002)_ = 3.82 × 10⁻^4^ cm^2^/Vs. The electron mobility is higher than the hole mobility for both orientations. These results are consistent with the previous research of Bruno Cucco et al. indicating that electron mobility is larger than hole mobility in halide perovskites [58]. Furthermore, there is little difference in electron mobility between the parallel and perpendicularly oriented samples. This suggests that the organic spacers in 4AMPPbI_4_ can be more favorable for electron transport than hole transport, referring to previously reported studies showing that electron confinement is weaker than hole confinement in 2D DJ perovskites with similar organic spacers [59].

While the difference in electron mobility between the two orientations is less pronounced, the perpendicularly oriented films nevertheless exhibit higher carrier mobility overall. This observation is further supported by conductive atomic force microscopy (C-AFM) analysis, which demonstrates improved charge transport in the perpendicularly oriented films at the microscopic level (Figure S9). By probing charge transport through localized regions (i.e., within a single grain), the C-AFM measurements confirm that the observed enhancement originates from the intrinsic properties of the perovskite film itself. The superior carrier transport observed in perpendicularly oriented 4AMPPbI_4_ under a vertical electric field can be attributed to the orientation of the 4AMP planes. In parallel-oriented films, these planes act as barriers to vertical charge transport. However, in the perpendicularly oriented films, the 4AMP planes do not significantly impede vertical carrier transport [60, 61]. Consequently, the carrier mobility for intralayer transport (perpendicular orientation) is higher than that for interlayer transport (parallel orientation). Conversely, the charge carrier mobility of parallel-oriented 4AMPPbI_4_ has been shown to be high in device structures, such as thin-film transistors, where horizontal charge carrier transport is dominant, as reported in the study by Zhu et al. Consequently, controlling crystal orientation represents a promising approach, enabling the tailored application of perovskites based on specific device architectures [62].

## Conclusion

This study developed a method to fabricate high-quality 4AMPPbI₄ perovskite thin films with controlled crystal orientations (parallel and perpendicular) by manipulating various crystallization parameters. Perpendicularly oriented films were obtained using a precursor solution with a low concentration of THTO, a chlorobenzene antisolvent to induce rapid nucleation at the air–liquid interface, and a stepwise annealing process. In contrast, parallel-oriented films were obtained using solutions with a high concentration of THTO and high-temperature annealing without an antisolvent. Direct and inverse photoelectron spectroscopy analysis revealed that the electronic structure of 4AMPPbI_4_ is orientation dependent. Notably, perpendicularly oriented films exhibited lower ionization energy and electron affinity compared to parallel-oriented films. The impact of orientation on charge transport was further investigated using single-carrier devices. Devices with perpendicularly oriented 4AMPPbI_4_ exhibited enhanced carrier mobility, attributed to the direct pathways for intralayer charge transport between the electrodes. These findings emphasize the importance of controlling crystal orientation for optimizing the performance of DJ phase 2D perovskite-based optoelectronic devices.

## Methods/experimental

### Materials

4-(aminomethyl)piperidine (4AMP, 96%), lead(II) oxide (PbO, 99.9%), hydroiodic acid (57 wt% in H_2_O, distilled, stabilized, 99.95%) and hypophosphorous acid solution (H_3_PO_2_, 50 wt % in H_2_O), dimethylformamide (DMF, 99.9%), dimethyl sulfoxide (DMSO, anhydrous, ≥ 99.9%), tetrahydrothiophene-1-oxide (THTO, 96%) and chlorobenzene (CB, 99.9%) were all purchased from Sigma-Aldrich.

### Synthesis of 4AMPPbI_4_ crystal powder

The protonated 4AMP precursor was obtained by directly mixing 1 mmol (114 mg) of 4AMP with 0.3 mL of hydroiodic acid. 1 mmol (223 mg) of PbO was dissolved in 5 mL of hydroiodic acid, followed by the addition of 0.5 mL of H_3_PO_2_. The solution was stirred at 130 ℃ for 30 min. Subsequently, the 4AMP precursor was added, and the solution was heated to 240 ℃ for 5 min. After cooling to room temperature, orange-red crystal powders of 4AMPPbI_4_ were obtained and characterized by powder X-ray diffraction (Figure S10). To prepare 0.5 M 4AMPPbI_4_ solutions, 0.5 mmol (416 mg) of 4AMPPbI_4_ powder was dissolved in 1 mL of DMF:THTO solvent mixtures with various ratios.

### Hole-only device (HOD) and electron-only device (EOD)

The HODs consisted of 4AMPPbI_4_ sandwiched between ITO and Al electrodes, with MoO_x_ layers thermally evaporated between the electrodes and the 4AMPPbI_4_ layer to facilitate hole injection and block electron injection (Al/MoO_x_/4AMPPbI_4_/MoO_x_/ITO). Similarly, EODs were fabricated with LiF layers inserted between the electrodes and the 4AMPPbI_4_ layer to facilitate electron injection and block hole injection (Al/LiF/4AMPPbI_4_/LiF/ITO).

### Characterization of 4AMPPbI_4_ powder

Powder X-ray diffraction (PXRD) patterns were recorded with a Rigaku Ultima IV powder diffractometer in a focused beam Bragg–Brentano geometry using Cu Kα (λ = 1.5406 Å) X-ray tube operated at 40 kV/40 mA. The phase purity of 4AMPPbI_4_ powder was confirmed by comparing the experimental data with simulations based on a refined crystal structure from Elbaz et al. [63] and a simulation algorithm implemented in VESTA program [64].

### Characterization of 4AMPPbI_4_ films

The surface morphology and cross-sections of 4AMPPbI_4_ thin films were characterized by atomic force microscopy (AFM, NX10, Park Systems) and field-emission scanning electron microscopy (FE-SEM, 7610F-Plus, JEOL). High-resolution XRD data for single crystals, including θ − 2θ scans, were collected using a Rigaku Smartlab diffractometer in a parallel beam geometry using Cu K_α_ (λ = 1.5406 Å, four-bounced Ge (220) monochromatized beam) X-ray tube operated at 45 kV/30 mA. The absorbance of the films was measured using UV/VIS spectrophotometer (V-650, JASCO).

Ultraviolet photoelectron spectroscopy (UPS) spectra were measured using a SPECS PHOIBOS 150 hemisphere analyzer and a He I_α_ (*hν* = 21.22 eV) UV discharge lamp. The secondary electron cutoff (SEC) was obtained with an applied bias of − 10 V during UPS measurements. Inverse photoelectron spectroscopy (IPES) spectra were measured in isochromat mode with a low-energy electron gun and a bandpass filter (SrF_2_–NaCl combination for 9.5 eV pass energy). The energy reference for both UPS and IPES was calibrated using clean Au. Core level spectra were measured by X-ray photoelectron spectroscopy (XPS, VersaProbe, Ulvac-Phi) using a monochromatic Al K_α_ (ℏω = 1486.6 eV) X-ray source. Current density–voltage (J–V) characteristics of perovskite films were measured using a Keithley 2400 source-measure unit (Tektronix Inc.)

## Supplementary Information

Supplementary materials 1.

## Data Availability

The data that support the findings of this study are available from the corresponding author upon reasonable request.

## References

[1] W.-J. Yin, T. Shi, Y. Yan, Adv. Mater. **26**, 4653 (2014). 10.1002/adma.201306281 24827122 10.1002/adma.201306281

[2] A.W. Faridi, M. Imran, G.H. Tariq, S. Ullah, S.F. Noor, S. Ansar, F. Sher, Ind. Eng. Chem. Res. **62**, 4494 (2023). 10.1021/acs.iecr.2c00416 36975768 10.1021/acs.iecr.2c00416 PMC10037322

[3] S. DeWolf, J. Holovsky, S.J. Moon, P. Loper, B. Niesen, M. Ledinsky, F.J. Haug, J.H. Yum, C. Ballif, J. Phys. Chem. Lett. **5**, 1035 (2014). 10.1021/jz500279b 26270984 10.1021/jz500279b

[4] L.M. Herz, ACS Energy Lett. **2**, 1539 (2017). 10.1021/acsenergylett.7b00276

[5] J. Lim, M. Kober-Czerny, Y.-H. Lin, J.M. Ball, N. Sakai, E.A. Duijnstee, M.J. Hong, J.G. Labram, B. Wenger, H.J. Snaith, Nat. Commun. **13**, 4021 (2022). 10.1038/s41467-022-31569-w 35859149 10.1038/s41467-022-31569-w PMC9300620

[6] K. Galkowski, A. Mitioglu, A. Miyata, P. Plochocka, O. Portugall, G.E. Eperon, J.T.-W. Wang, T. Stergiopoulos, S.D. Stranks, H.J. Snaith, R.J. Nicholas, Energy Environ. Sci. **9**, 962 (2016). 10.1039/C5EE03435C

[7] L.M. Herz, Annu. Rev. Phys. Chem. **67**, 65 (2016). 10.1146/annurev-physchem-040215-112222 26980309 10.1146/annurev-physchem-040215-112222

[8] Y. Dong, T. Qiao, D. Kim, D. Parobek, D. Rossi, D.H. Son, Nano Lett. **18**, 3716 (2018). 10.1021/acs.nanolett.8b00861 29727576 10.1021/acs.nanolett.8b00861

[9] Y. Ding, Z. Zhang, S. Toso, I. Gushchina, V. Trepalin, K. Shi, J.W. Peng, M. Kuno, J. Am. Chem. Soc. **145**, 6362 (2023). 10.1021/jacs.2c13527 36881007 10.1021/jacs.2c13527

[10] B.J. Bohn, Y. Tong, M. Gramlich, M.L. Lai, M. Döblinger, K. Wang, R.L.Z. Hoye, P.M. Buschbaum, S.D. Stranks, A.S. Urban, L. Polavarapu, J. Feldmann, Nano Lett. **18**, 5231 (2018). 10.1021/acs.nanolett.8b02190 29990435 10.1021/acs.nanolett.8b02190

[11] J.Y. Kim, J.-W. Lee, H.S. Jung, H. Shin, N.-G. Park, Chem. Rev. **120**, 7867 (2020). 10.1021/acs.chemrev.0c00107 32786671 10.1021/acs.chemrev.0c00107

[12] G. Yang, Z. Ren, K. Liu, M. Qin, W. Deng, H. Zhang, H. Wang, J. Liang, F. Ye, Q. Liang, H. Yin, Y. Chen, Y. Zhuang, S. Li, B. Gao, J. Wang, T. Shi, X. Wang, X. Lu, H. Wu, J. Hou, D. Lei, S.K. So, Y. Yang, G. Li, Nat. Photon. **15**, 681 (2021). 10.1038/s41566-021-00829-4

[13] I.J. Park, H.J. An, Y. Chang, J.Y. Kim, Nano Converg. **10**, 22 (2023). 10.1186/s40580-023-00374-6 37209284 10.1186/s40580-023-00374-6 PMC10199996

[14] W. Zheng, Q. Wan, Q. Zhang, M. Liu, C. Zhang, B. Wang, L. Kong, L. Li, Nanoscale **12**, 8711 (2020). 10.1039/D0NR01681K 32285067 10.1039/d0nr01681k

[15] K. Lê, N. Heshmati, S. Mathur, Nano Converg. **10**, 47 (2023). 10.1186/s40580-023-00395-1 37831205 10.1186/s40580-023-00395-1 PMC10575846

[16] B.R. Sutherland, E.H. Sargent, Nat. Photon. **10**, 295 (2016). 10.1038/NPHOTON.2016.62

[17] M.I. Saidaminov, V. Adinolfi, R. Comin, A.L. Abdelhady, W. Peng, I. Dursun, M. Yuan, S. Hoogland, E.H. Sargent, O.M. Bakr, Nat. Commun. **6**, 8724 (2015). 10.1038/ncomms9724 26548941 10.1038/ncomms9724 PMC4667636

[18] J. Miao, F. Zhang, J. Mater. Chem. C. **7**, 1741 (2019). 10.1039/c8tc06089d

[19] D. Kim, T. Yun, S. An, C.-L. Lee, Nano Converg. **11**, 4 (2024). 10.1186/s40580-024-00412-x 38279984 10.1186/s40580-024-00412-x PMC10821855

[20] Y.-W. Jang, S. Lee, K.M. Yeom, K. Jeong, K. Choi, M. Choi, J.H. Noh, Nat. Energy **6**, 63 (2021). 10.1038/s41560-020-00749-7

[21] H. Tsai, W. Nie, J.-C. Blancon, C.C. Stoumpos, R. Asadpour, B. Harutyunyan, A.J. Neukirch, R. Verduzco, J.J. Crochet, S. Tretiak, L. Pedesseau, J. Even, M.A. Alam, G. Gupta, J. Lou, P.M. Ajayan, M.J. Bedzyk, M.G. Kanatzidis, A.D. Mohite, Nature **536**, 312 (2016). 10.1038/nature18306 27383783 10.1038/nature18306

[22] H. Sirringhaus, P.J. Brown, R.H. Friend, M.M. Nielsen, K. Bechgaard, B.M.W. Langeveld-Voss, A.J.H. Spiering, R.A.J. Janssen, E.W. Meijer, P. Herwig, D.M. de Leeuw, Nature **401**, 685 (1999). 10.1038/44359

[23] S. Duhm, G. Heimel, I. Salzmann, H. Glowatzki, R.L. Johnson, A. Vollmer, J.P. Rabe, N. Koch, Nat. Mat. **7**, 326 (2008). 10.1038/nmat2119 10.1038/nmat2119 18264103

[24] N. Koch, I. Salzmann, R.L. Johnson, J. Pflaum, R. Friedlein, J.P. Rabe, Org. Electron. **7**, 537 (2006). 10.1016/j.orgel.2006.07.010

[25] J. Ivanco, T. Haber, J.R. Krenn, F.P. Netzer, R. Resel, M.G. Ramsey, Surf. Sci. **601**, 178 (2007). 10.1016/j.susc.2006.09.020

[26] X. Zhang, G. Wu, W. Fu, M. Qin, W. Yang, J. Yan, Z. Zhang, X. Lu, H. Chen, Adv. Energy Mater. **8**, 1702498 (2018). 10.1002/aenm.201702498

[27] A.Z. Chen, M. Shiu, X. Deng, M. Mahmoud, D. Zhang, B.J. Foley, S.-H. Lee, G. Giri, J.J. Choi, Chem. Mater. **31**, 1336 (2019). 10.1021/acs.chemmater.8b04531

[28] Y. Liao, H. Liu, W. Zhou, D. Yang, Y. Shang, Z. Shi, B. Li, X. Jiang, L. Zhang, L.N. Quan, R. Quintero-Bermudez, B.R. Sutherland, Q. Mi, E.H. Sargent, Z. Ning, J. Am. Chem. Soc. **139**, 6693 (2017). 10.1021/jacs.7b01815 28438016 10.1021/jacs.7b01815

[29] W. Zhang, Z. Liu, L. Zhang, H. Wang, C. Jiang, X. Wu, C. Li, S. Yue, R. Yang, H. Zhang, J. Zhang, X. Liu, Y. Zhang, H. Zhou, Nat. Commun. **15**, 5709 (2024). 10.1038/s41467-024-50018-4 38977696 10.1038/s41467-024-50018-4 PMC11231157

[30] L. Cheng, Z. Liu, S. Li, Y. Zhai, X. Wang, Z. Qiao, Q. Xu, K. Meng, Z. Zhu, G. Chen, Angew. Chem. Int. Ed. **60**, 856 (2021). 10.1002/ange.202006970 10.1002/anie.202006970 33021033

[31] Y. Shang, Y. Liao, Q. Wei, Z. Wang, B. Xiang, Y. Ke, W. Liu, Z. Ning, Sci. Adv. **5**, 8072 (2019). 10.1126/sciadv.aaw8072 10.1126/sciadv.aaw8072 PMC6697432 31453330

[32] S. Ahmad, P. Fu, S. Yu, Q. Yang, X. Liu, X. Wang, X. Wang, X. Guo, C. Li, Joule. **3**, 794 (2019). 10.1016/j.joule.2018.11.026

[33] L. Mao, W. Ke, L. Pedesseau, Y. Wu, C. Katan, J. Even, M.R. Wasielewski, C.C. Stoumpos, M.G. Kanatzidis, J. Am. Chem. Soc. **140**, 3775 (2018). 10.1021/jacs.8b00542 29465246 10.1021/jacs.8b00542

[34] I.-H. Park, Q. Zhang, K.C. Kwon, Z. Zhu, W. Yu, K. Leng, D. Giovanni, H.S. Choi, I. Abdelwahab, Q.-H. Xu, T.C. Sum, K.P. Loh, J. Am. Chem. Soc. **141**, 15972 (2019). 10.1021/jacs.9b07776 31522501 10.1021/jacs.9b07776

[35] J. Yin, R. Naphade, P. Maity, L.G. Arzaluz, D. Almalawi, I.S. Roqan, J.L. Brédas, O.M. Bakr, O.F. Mohammed, Nat. Commun. **12**, 3995 (2021). 10.1038/s41467-021-24258-7 34183646 10.1038/s41467-021-24258-7 PMC8239041

[36] L. Ji, T. Zhang, Y. Wang, P. Zhang, D. Liu, Z. Chen, S. Li, Nanoscale Res. Lett. **12**, 367 (2017). 10.1186/s11671-017-2117-6 28535603 10.1186/s11671-017-2117-6 PMC5440423

[37] C. Wang, Y. Zhao, T. Ma, Y. An, R. He, J. Zhu, C. Chen, S. Ren, F. Fu, D. Zhao, X. Li, Nat. Energy **7**, 744 (2022). 10.1038/s41560-022-01076-9

[38] L.-C. Chen, J.-R. Wu, Z.-L. Tseng, C.-C. Chen, S.H. Chang, J.-K. Huang, K.-L. Lee, H.-M. Cheng, Materials. **9**, 747 (2016). 10.3390/ma9090747 28773874 10.3390/ma9090747 PMC5457094

[39] F. Guo, S. Qiu, J. Hu, H. Wang, B. Cai, J. Li, X. Yuan, X. Liu, K. Forberich, C.J. Brabec, Y. Mai, Adv. Sci. **6**, 1901067 (2019). 10.1002/advs.201901067 10.1002/advs.201901067 PMC6724353 31508290

[40] Z. Wang, X. Liu, Y. Lin, Y. Liao, Q. Wei, H. Chen, J. Qiu, Y. Chen, Y. Zheng, J. Mater. Chem. A. **7**, 2773 (2019). 10.1039/c8ta09855g

[41] B.J. Foley, J. Girard, B.A. Sorenson, A.Z. Chen, J.S. Niezgoda, M.R. Alpert, A.F. Harper, D.-M. Smilgies, P. Clancy, W.A. Saidie, J.J. Choi, J. Mater. Chem. A. **5**, 113 (2017). 10.1039/c6ta07671h

[42] N. Giesbrecht, J. Schlipf, I. Grill, P. Rieder, V. Dyakonov, T. Bein, A. Hartschuh, P.M. Buschbaum, P. Docampo, J. Mater. Chem. A. **6**, 4822 (2018). 10.1039/C7TA11190H

[43] A.Z. Chen, M. Shiu, J.H. Ma, M.R. Alpert, D. Zhang, B.J. Foley, D.-M. Smilgies, S.-H. Lee, J.J. Choi, Nature Commun. **9**, 1336 (2018). 10.1038/s41467-018-03757-0 29626205 10.1038/s41467-018-03757-0 PMC5889398

[44] D. Zheng, F. Raffin, P. Volovitch, T. Pauporté, Nat. Commun. **13**, 6655 (2022). 10.1038/s41467-022-34332-3 36333344 10.1038/s41467-022-34332-3 PMC9636165

[45] Y. Chen, J. Hu, Z. Xu, Z. Jiang, S. Chen, B. Xu, X. Xiao, X. Liu, K. Forberich, C.J. Brabec, Y. Mai, F. Guo, Adv. Funct. Mater. **32**, 2112146 (2022). 10.1002/adfm.202112146

[46] Y. Hu, L.M. Spies, D.A. Álvarez, P. Mocherla, H. Jones, J. Hanisch, T. Bein, P.R.F. Barnes, P. Docampo, J. Mater. Chem. A. **6**, 22215 (2018). 10.1039/c8ta05475d

[47] M. Rahil, R.M. Ansari, C. Prakash, S.S. Islam, A. Dixit, S. Ahmad, Sci. Rep. **12**, 2176 (2022). 10.1038/s41598-022-06108-8 35140250 10.1038/s41598-022-06108-8 PMC8828857

[48] Y. Chen, Y. Sun, J. Peng, J. Tang, K. Zheng, Z. Liang, Adv. Mater. **30**, 1703487 (2018). 10.1002/adma.201703487 10.1002/adma.201703487 29028138

[49] D.H. Cao, C.C. Stoumpos, O.K. Farha, J.T. Hupp, M.G. Kanatzidis, J. Am. Chem. Soc. **137**, 7843 (2015). 10.1021/jacs.5b03796 26020457 10.1021/jacs.5b03796

[50] K.R. Hansen, C.Y. Wong, C.E. McClure, B. Romrell, L. Flannery, D. Powell, K. Garden, A. Berzansky, M. Eggleston, D.J. King, C.M. Shirley, M.C. Beard, W. Nie, A. Schleife, J.S. Colton, L.W. Brooks, Matter **6**, 3463 (2023). 10.1016/j.matt.2023.07.004

[51] R. Chakraborty, A. Nag, J. Phys. Chem. C **124**, 16177 (2020). 10.1021/acs.jpcc.0c04284

[52] M. Baranowski, P. Plochocka, Adv. Energy Mater. **10**, 1903659 (2020). 10.1002/aenm.201903659

[53] A. Kahn, Mater. Horiz. **3**, 7 (2016). 10.1039/c5mh00160a

[54] Q. Dong, Y. Fang, Y. Shao, P. Mulligan, J. Qiu, L. Cao, J. Huang, Science **347**, 967 (2015). 10.1126/science.aaa5760 25636799 10.1126/science.aaa5760

[55] F. Cai, Y. Yan, J. Yao, P. Wang, H. Wang, R. S. Gurney, D. Liu., T. Wang, Adv. Funct. Mater. **28**, 1801985 (2018), 10.1002/adfm.201801985

[56] B. Chen, R. Yu, G. Xing, Y. Wang, W. Wang, Y. Chen, X. Xu, Q. Zhao, ACS Energy Lett. **9**, 226 (2024). 10.1021/acsenergylett.3c02069

[57] H. Takagi, H. Kunugita, K. Ema, Phys. Rev. B **87**, 125421 (2013). 10.1103/PhysRevB.87.125421

[58] B. Cucco, J. Leveillee, V.-A. Ha, J. Even, M. Kepenekian, F. Giustino, G. Volonakis, PRX Energy. **3**, 023012 (2024). 10.1103/PRXEnergy.3.023012

[59] N.F.N.A. Yami, A. Ramli, W.I. Nawawi, S. Sepeai, S.D. Safian, N.H.M. Zaki, M.F.M. Taib, O.H. Hassan, A.M.M. Ali, Emergent Mater. **6**, 999 (2023). 10.1007/s42247-023-00484-1

[60] G. Uzurano, N. Kuwahara, T. Saito, K. Abe, S. Miyake, D. Hishida, Y. Takeoka, A. Fujii, and M. Ozaki, Appl. Phys. Express, **16**, 041005 (2023), 10.35848/1882-0786/accd44

[61] K. Yang, Y. Kang, S. Meng, J. Zhang, W. Ma, Nano Lett. **24**, 5057 (2024). 10.1021/acs.nanolett.4c00851 10.1021/acs.nanolett.4c00851 38587481

[62] H. Zhu, W. Yang, Y. Reo, G. Zheng, S. Bai, A. Liu, Y.-Y. Noh, Nature Electronics. **6**, 650 (2023). 10.1038/s41928-023-01019-6

[63] G.A. Elbaz, D.B. Straus, O.E. Semonin, T.D. Hull, D.W. Paley, P. Kim, J.S. Owen, C.R. Kagan, X. Roy, Nano Lett. **17**, 1727 (2017). 10.1021/acs.nanolett.6b05022 28240556 10.1021/acs.nanolett.6b05022

[64] K. Momma, F. Izumi, J. Appl. Cryst. **44**, 1272 (2011). 10.1107/S0021889811038970

