# Supplementary material for: Tuning electronic structure and carrier transport properties through crystal orientation control in two-dimensional Dion-Jacobson phase perovskites
Source: Nano Converg. 2025 Jan 13;12:1. doi: 10.1186/s40580-024-00473-y (PMC11730049; doi:10.1186/s40580-024-00473-y)
Supplement: Supplementary file 1 — Supplementary materials 1. [file 40580_2024_473_MOESM1_ESM.docx]

[Supplementary Material]

Tuning Electronic Structure and Carrier Transport Properties through Crystal Orientation Control in Two-Dimensional Dion-Jacobson Phase Perovskites

Byunggeol Kim^a,†^ , Jeehong Park ^a,†^, Donghee Kang ^a^, Na Eun Jung ^a^, Kitae Kim^a,b^, Hongsun Ryu^c^, Joon Ik Jang^c^, Soohyung Park^b^, and Yeonjin Yi ^a,^*

^a^Department of Physics, Yonsei University, Seoul 03722, Republic of Korea

^b^Advanced Analysis & Data Center, Korea Institute of Science and Technology (KIST), Seoul 02792, Republic of Korea

^c^Department of Physics, Sogang University, Seoul, 04107, Republic of Korea

† These authors contributed equally to this work as first authors.

*To whom correspondence should be addressed

Tel: +82 2 2123 5612; Fax: +82 2 392 1592

E-mail: [yeonjin@yonsei.ac.kr](mailto:yeonjin@yonsei.ac.kr)

**
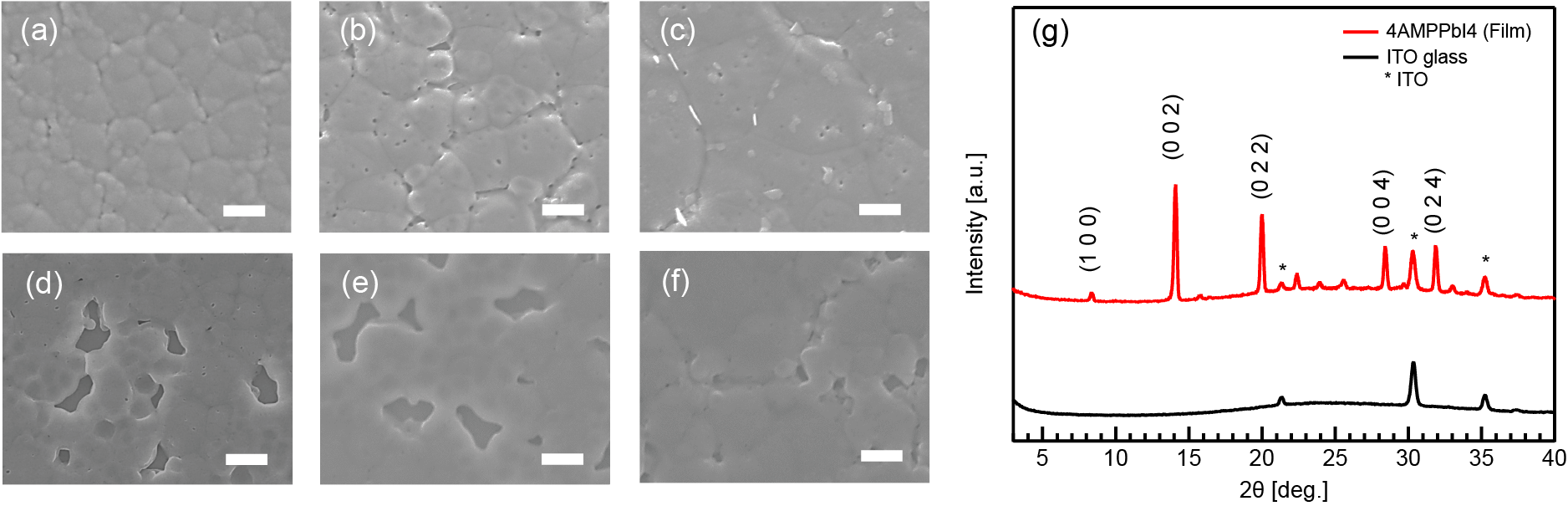
**

Figure S1. SEM images of perovskite thin films (scale bar = 1 μm) prepared with: annealing temperatures of (a) 100℃, (b) 150℃, (c) 170℃, and solvent ratios (DMF:DMSO) of (d) 4:1, (e) 6:1, (f) 10:1 (DMF:DMSO). (g) XRD pattern of the perovskite thin film prepared with a solvent ratio (DMF:DMSO) of 4:1.


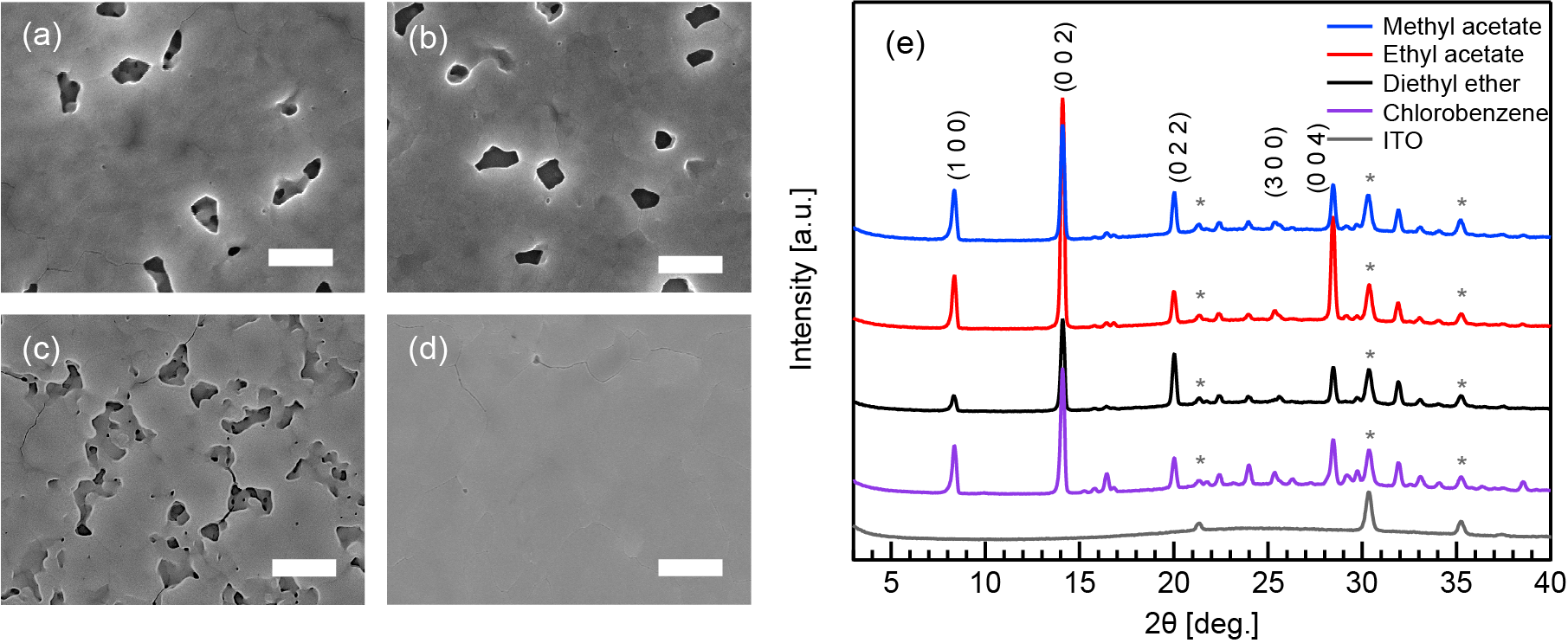


Figure S2. SEM images (scale bar = 1 μm) of perovskite thin films prepared with various antisolvents, (a) Methyl acetate (b) Ethyl acetate (c) Diethyl ether (d) Chlorobenzene. (e) XRD patterns of the corresponding films.


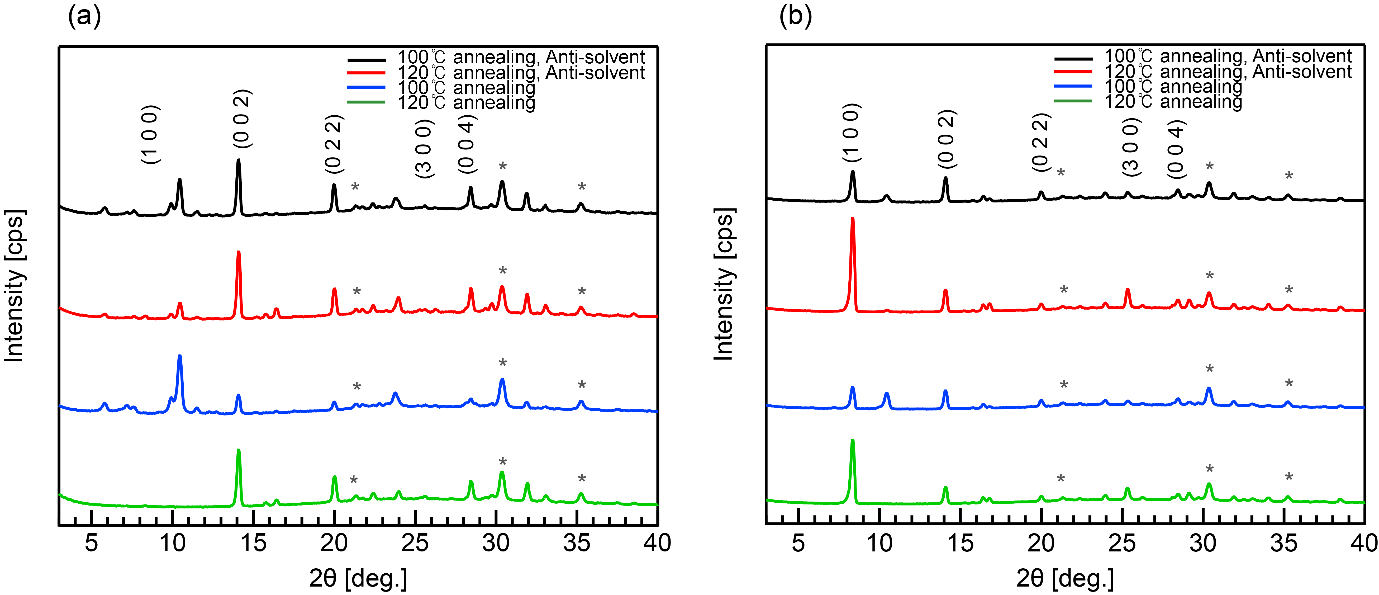


Figure S3. XRD patterns of perovskite films prepared with varying annealing temperatures and antisolvents using solvent ratios (DMF:THTO) of (a) 10:1 and (b) 4:1.


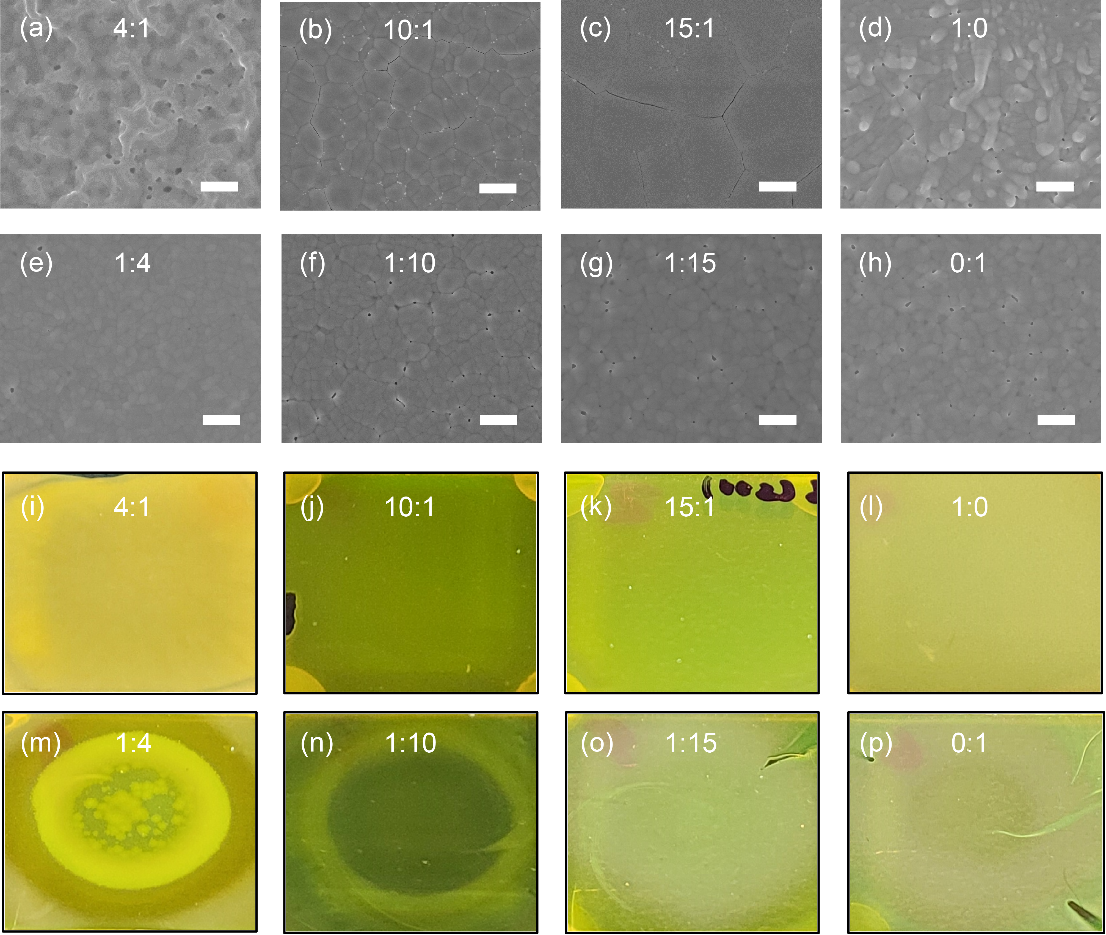


Figure S4. Surface morphologies of films fabricated by the M1 process (with antisolvent) using perovskite solutions with varying DMF:THTO ratios. SEM images (scale bar = 1 μm) of the ratios of (a) 4:1, (b) 10:1, (c) 15:1, (d) 1:0, (e) 1:4, (f) 1:10 (g) 1:15, and (h) 0:1; Optical images of the ratios of (i) 4:1, (j) 10:1, (k) 15:1, (l) 1:0, (m) 1:4, (n) 1:10 (o) 1:15, and (p) 0:1.


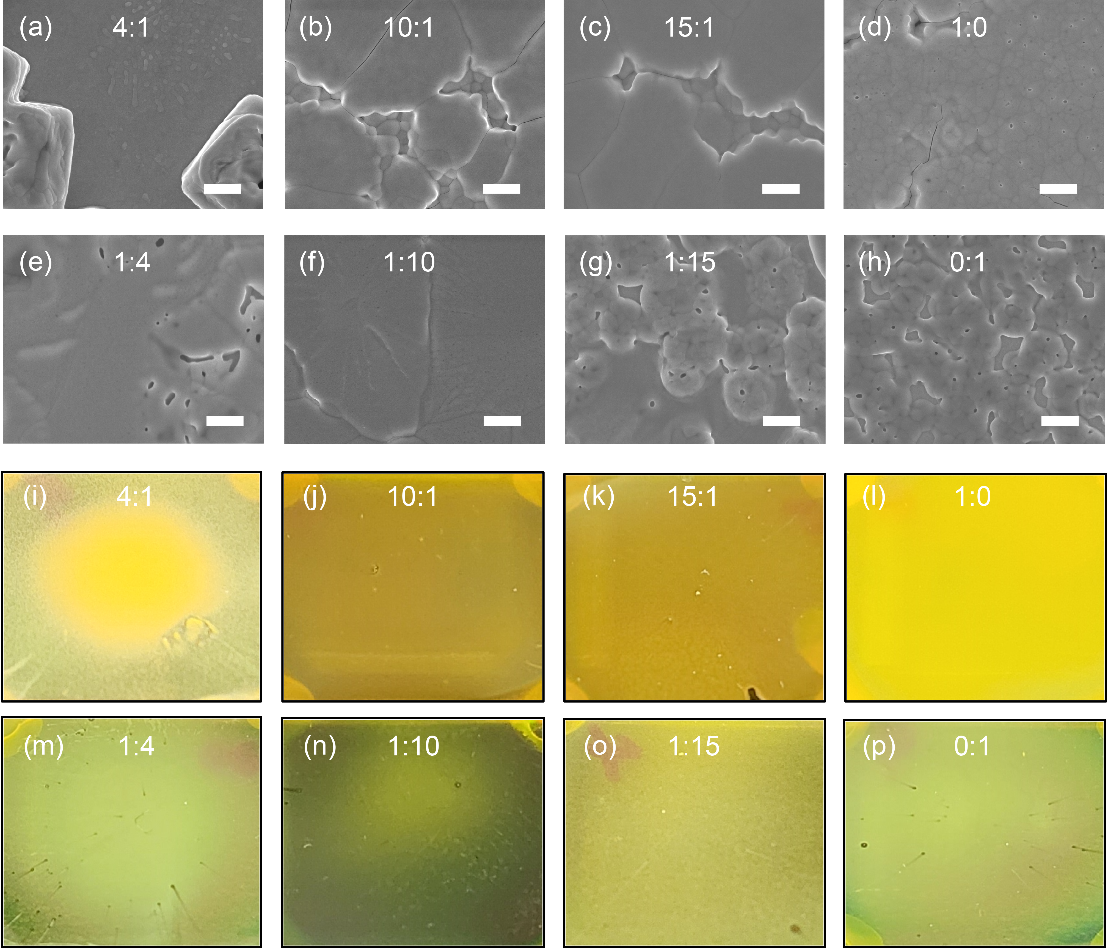


Figure S5. Surface morphologies of films fabricated by the M2 process using perovskite solutions with varying DMF:THTO ratios. SEM images (scale bar = 1 μm) of the ratios of (a) 4:1, (b) 10:1, (c) 15:1, (d) 1:0, (e) 1:4, (f) 1:10 (g) 1:15, and (h) 0:1; Optical images of the ratios of (i) 4:1, (j) 10:1, (k) 15:1, (l) 1:0, (m) 1:4, (n) 1:10 (o) 1:15, and (p) 0:1.


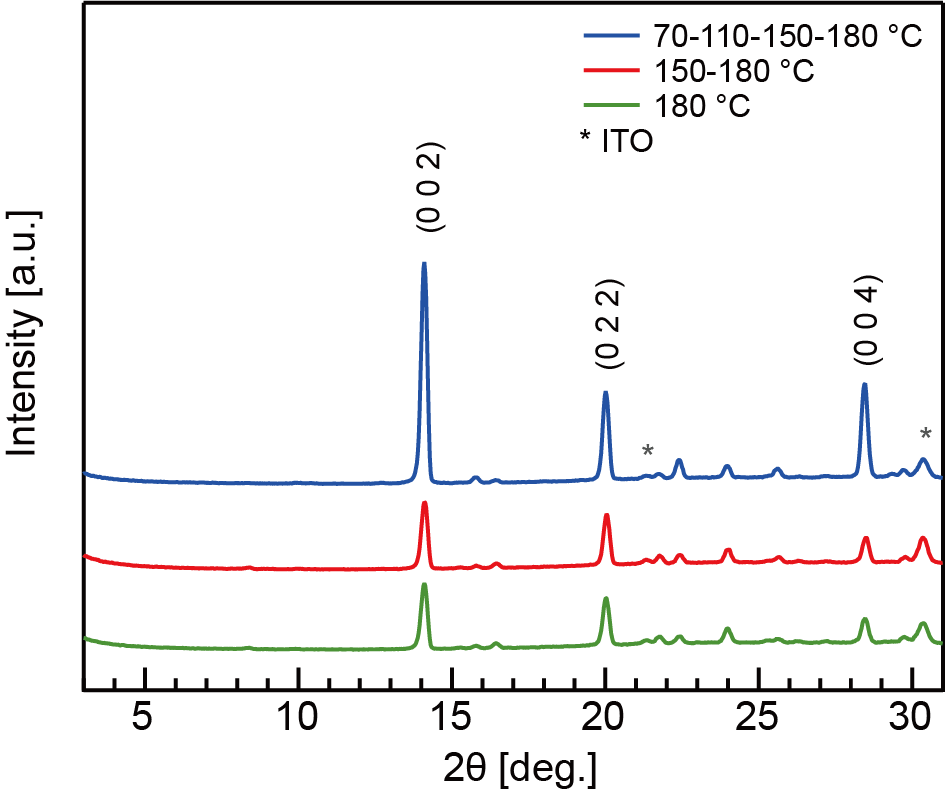


Figure S6. XRD patterns of films prepared with different annealing procedures: annealing at 180℃ (green line), annealing at 150 ℃ then 180 ℃ (red line), and annealing stepwise at 70 ℃, 110 ℃, 150 ℃, and finally 180 ℃ (blue line).


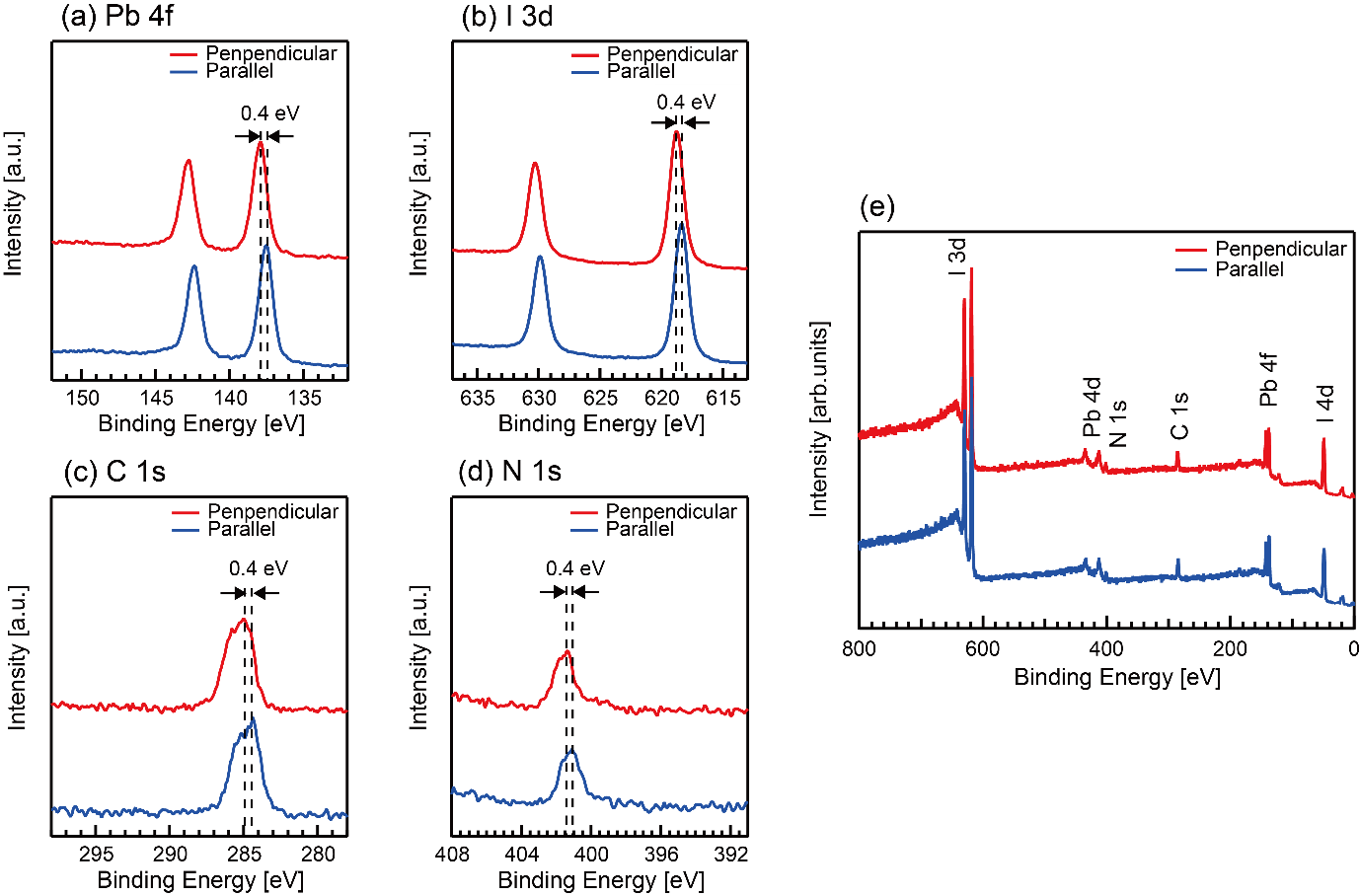


Figure S7. Core-level XRS spectra of 4AMPPbI_4_ films: (a) Pb 4f, (b) I 3d, (c) C 1s, (d) N 1s, and (e) survey scan.


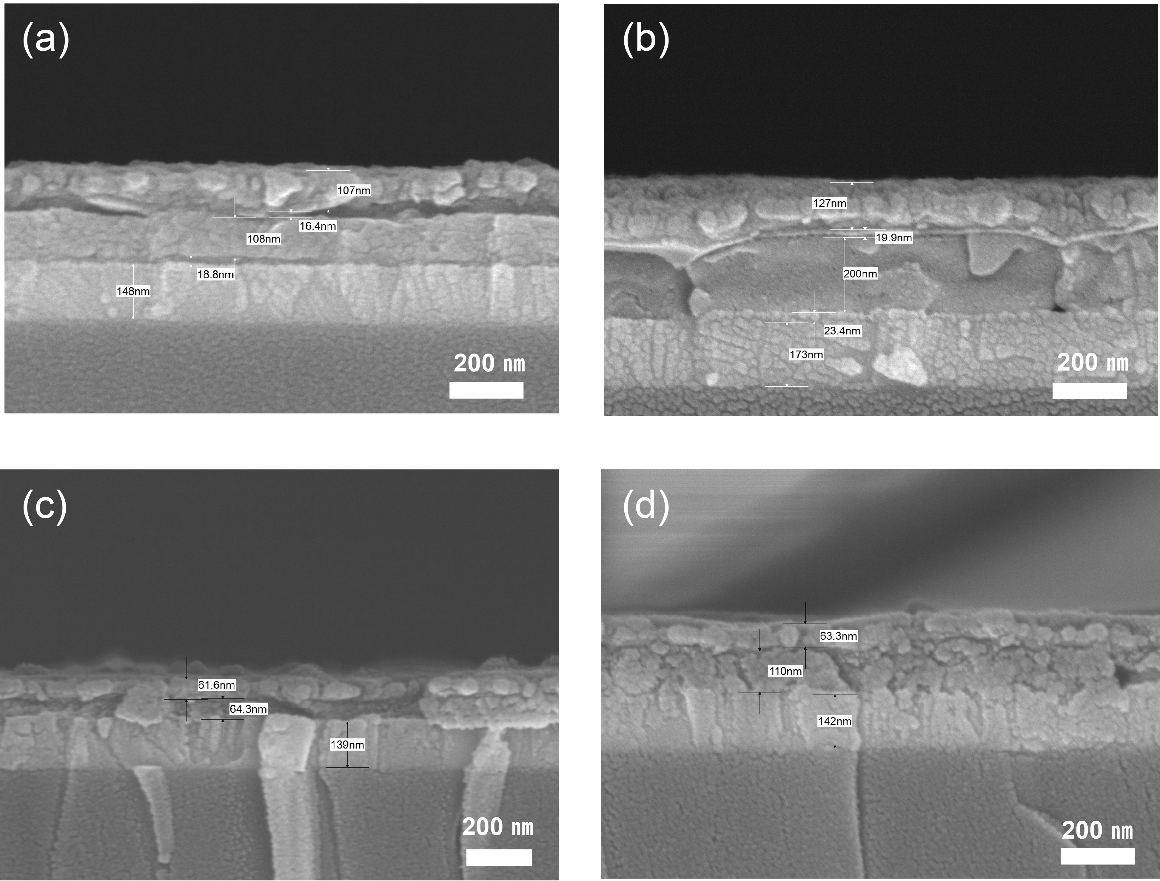


Figure S8. SEM cross-sectional images of the single-carrier devices: hole-only devices with (a) parallel and (b) perpendicular orientation; Electron-only devices with (c) parallel and (d) perpendicular orientation.

|  | E_b_ (meV) | ε_w_ | ε_b_ | L_w_ (Å) | L_b_ (Å) | ε_r_ |
| --- | --- | --- | --- | --- | --- | --- |
| 4AMPPbI_4_ | 90^a^ | 6.1^b^ | 5.1 | 6.3^c^ | 4.2^c^ | 5.7 |
| ^a^Experimented in this paper  ^b^Mateusz Dyksik [4]  ^c^Mao et al. [3] | | | | | | |

The dielectric constant of 2D perovskite was estimated based on the model proposed by Bing Chen et al [1], which describe the exciton binding energy (*E_b_*) for dimensionality reduction from 3D to 2D, considering dielectric confinement effects:

Table S1. Summary of parameters for 4AMPPbI_4_.

$E_{b,2D}\approx4{\cdot\left( \frac{\varepsilon_{w}}{\varepsilon_{b}} \right)}^{2}\cdot E_{b,3D}$

where *E_b,2D_* is the exciton binding energy of 4AMPPbI_4_ (90 meV) determined experimentally in this work, and *ε_b_* and *ε_w_* are the dielectric constants of the organic barrier and inorganic well in 4AMPPbI_4_, respectively. To calculate the dielectric constant ratio (*ε_w_*/*ε_b_*) of the 4AMPPbI_4_, the well-known exciton binding energy of MAPbI_3_ (*E_b,3D_* = 16 meV) was used [2].

The relative dielectric constant (*ε_r_*) can be evaluated as,

$$\varepsilon_{r}= \frac{\varepsilon_{w}L_{w}+ \varepsilon_{b}L_{b}}{L_{w}+ L_{b}}$$

where *L_w_* is the length of the inorganic well, approximated by the thickness of a [PbI_6_]^4-^ layer (6.3 Å), and *L_b_* is the distance between two [PbI_6_]^4-^octahedra, defining the length of the 4AMP organic barrier (4.2 Å). The values were obtained from the data reported by Lingling Mao et al [3]. Using these parameters, the *ε_r_* of 4AMPPbI_4_ was estimated to be 5.7 eV. This value is reasonable considering that 4AMPPbI_4_ has a shorter interlayer distance than PEA_2_PbI_4_ due to hydrogen bonding between the organic spacer and the inorganic layer [4].


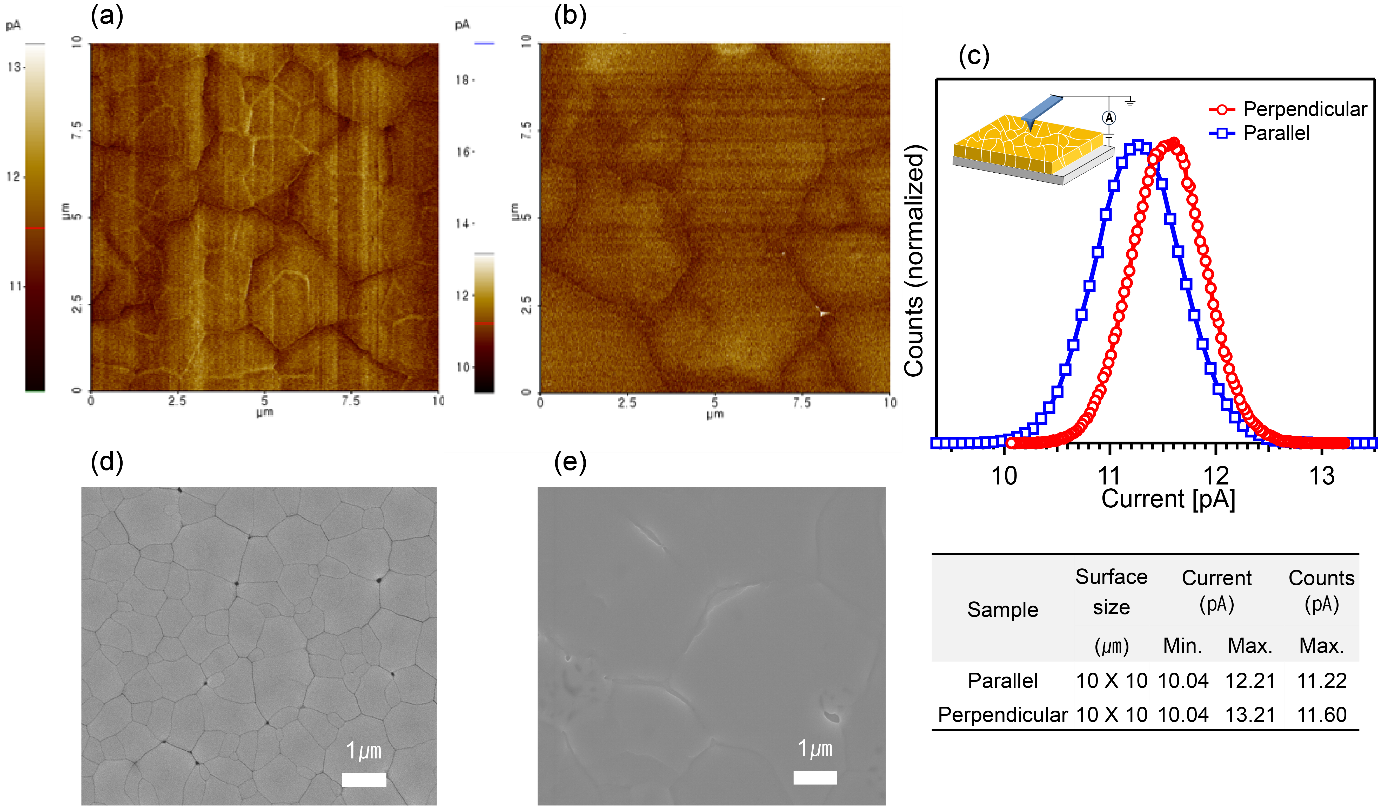


Figure S9. C-AFM measurements of (a) perpendicularly and (b) parallel oriented 4AMPPbI_4_ films. (c) Relative current comparison between perpendicular and parallel orientations. Corresponding SEM images of (d) perpendicular and (e) parallel orientation.


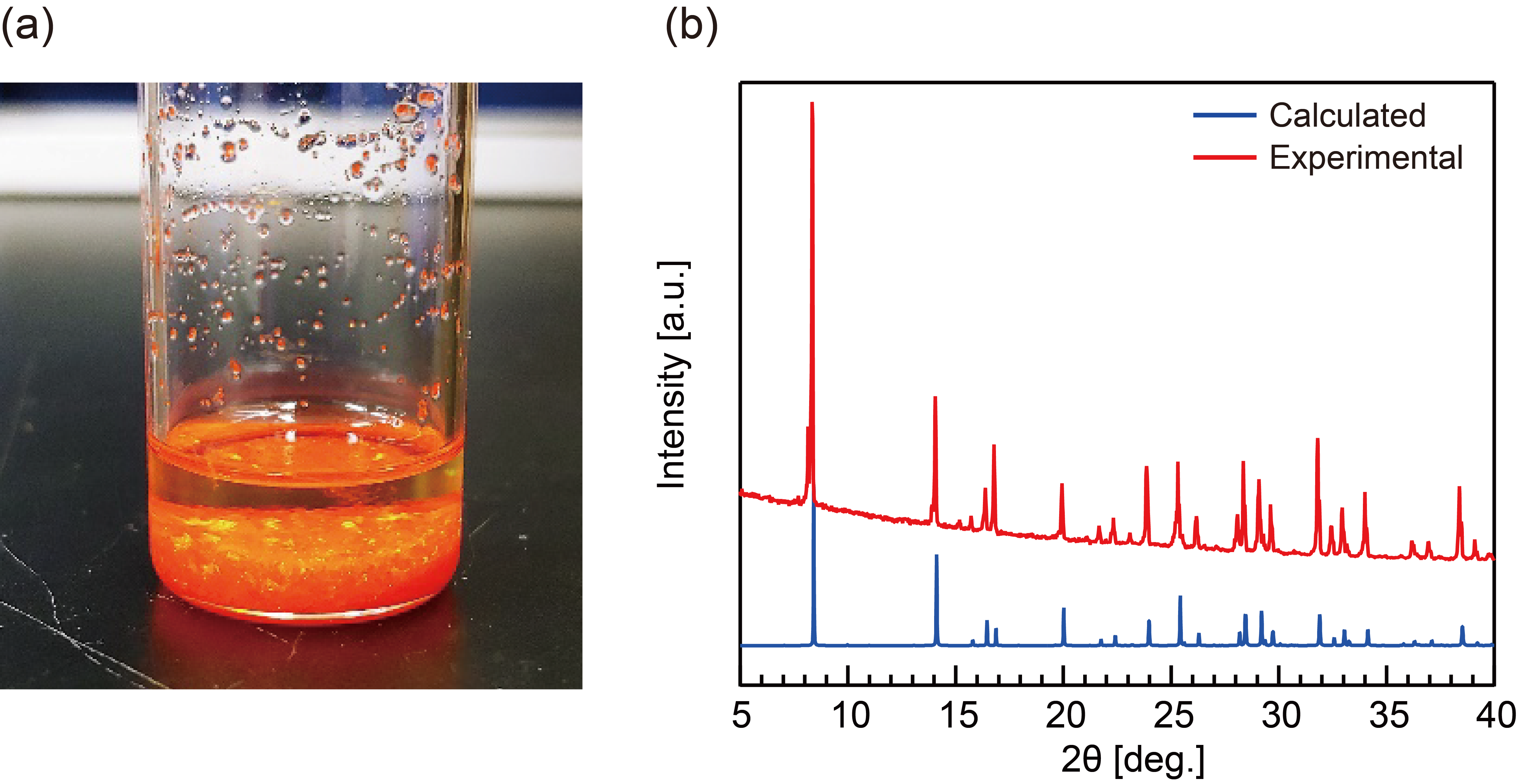


Figure S10. (a) Optical image of the 2D Dion-Jacobson 4AMPPbI_4_ perovskite (n=1) and (b) its powder XRD patterns (calculated^1^ and experimental).

**References**

1. Bing Chen, Rongrong Yu, Guansheng Xing, Yulong Wang, Wenlong Wang, Ya Chen, Xiuwen Xu, and Qiang Zhao, ACS Energy Lett., **9**, 226 (2024)

2. Kameron R. Hansen, Cindy Y. Wong, C. Emma McClure, Blake Romrell, Laura Flannery, Daniel Powell, Kelsey Garden, Alex Berzansky, Michele Eggleston, Daniel J. King, Carter M. Shirley, Matthew C. Beard, Wanyi Nie, André Schleife, John S. Colton, and Luisa Whittaker-Brooks, Matter **6**, 3463 (2023)

3. Lingling Mao, Weijun Ke, Laurent Pedesseau, Yilei Wu, Claudine Katan, Jacky Even, Michael R. Wasielewski, Constantinos C. Stoumpos and Mercouri G. Kanatzidis, J. Am. Chem. Soc., **140**, 3775 (2018)

4. Mateusz Dyksik, Int. J. Mol. Sci., **23**, 12531 (2022)
